# Validity and reliability of a novel immunosuppressive adverse effects scoring system in renal transplant recipients

**DOI:** 10.1186/1471-2369-15-88

**Published:** 2014-06-12

**Authors:** Calvin J Meaney, Ziad Arabi, Rocco C Venuto, Joseph D Consiglio, Gregory E Wilding, Kathleen M Tornatore

**Affiliations:** 1 Immunosuppressive Pharmacology Research Program, Translational Pharmacology Core, NYS Center of Excellence in Bioinformatics and Life Sciences, 701 Ellicott Street, Buffalo, New York 14203, USA; 2 Pharmacy Practice, School of Pharmacy and Pharmaceutical Sciences, University at Buffalo, 201 Kapoor Hall, Buffalo, New York 14214, USA; 3 Medicine, Nephrology Division; School of Medicine and Biomedical Sciences, University at Buffalo, Buffalo, NY, USA; 4 Biostatistics; School of Public Health, University at Buffalo, Buffalo, NY, USA; 5 Erie County Medical Center, Buffalo, NY, USA

**Keywords:** Immunosuppressive agents, Adverse effects, Renal transplantation, Calcineurin inhibitors, Mycophenolic acid, Tacrolimus, Cyclosporine

## Abstract

**Background:**

After renal transplantation, many patients experience adverse effects from maintenance immunosuppressive drugs. When these adverse effects occur, patient adherence with immunosuppression may be reduced and impact allograft survival. If these adverse effects could be prospectively monitored in an objective manner and possibly prevented, adherence to immunosuppressive regimens could be optimized and allograft survival improved. Prospective, standardized clinical approaches to assess immunosuppressive adverse effects by health care providers are limited. Therefore, we developed and evaluated the application, reliability and validity of a novel adverse effects scoring system in renal transplant recipients receiving calcineurin inhibitor (cyclosporine or tacrolimus) and mycophenolic acid based immunosuppressive therapy.

**Methods:**

The scoring system included 18 non-renal adverse effects organized into gastrointestinal, central nervous system and aesthetic domains developed by a multidisciplinary physician group. Nephrologists employed this standardized adverse effect evaluation in stable renal transplant patients using physical exam, review of systems, recent laboratory results, and medication adherence assessment during a clinic visit. Stable renal transplant recipients in two clinical studies were evaluated and received immunosuppressive regimens comprised of either cyclosporine or tacrolimus with mycophenolic acid. Face, content, and construct validity were assessed to document these adverse effect evaluations. Inter-rater reliability was determined using the Kappa statistic and intra-class correlation.

**Results:**

A total of 58 renal transplant recipients were assessed using the adverse effects scoring system confirming face validity. Nephrologists (subject matter experts) rated the 18 adverse effects as: 3.1 ± 0.75 out of 4 (maximum) regarding clinical importance to verify content validity. The adverse effects scoring system distinguished 1.75-fold increased gastrointestinal adverse effects (p = 0.008) in renal transplant recipients receiving tacrolimus and mycophenolic acid compared to the cyclosporine regimen. This finding demonstrated construct validity. Intra-class correlation was 0.81 (95% confidence interval: 0.65-0.90) and Kappa statistic of 0.68 ± 0.25 for all 18 adverse effects and verified substantial inter-rater reliability.

**Conclusions:**

This immunosuppressive adverse effects scoring system in stable renal transplant recipients was evaluated and substantiated face, content and construct validity with inter-rater reliability. The scoring system may facilitate prospective, standardized clinical monitoring of immunosuppressive adverse drug effects in stable renal transplant recipients and improve medication adherence.

## Background

Renal transplantation is the preferred intervention for patients with end-stage renal disease due to decreased patient morbidity and mortality compared to dialysis [1-3]. Renal transplant recipients are treated with combination immunosuppression consisting of calcineurin inhibitors such as cyclosporine or tacrolimus and mycophenolic acid to prevent allograft rejection [4]. However, these medications are associated with significant adverse effects which increase patient morbidity and decrease medication adherence [4-14]. Patient non-adherence with immunosuppressive medications has increased as a result of mild to moderate adverse effects which are not consistently documented during outpatient clinic appointments. This may impact acute rejection and long-term allograft survival post-transplant [15-17]. Prospective clinical monitoring of immunosuppressive adverse effects is critical to prevent post-transplant complications and improve patient safety outcomes.

Routine clinical assessment of immunosuppressive adverse effects has not included standardized, objective rating guides with quantitation of severity. Therefore, adverse effect evaluation post-transplant is variable among clinicians resulting in inconsistent reporting and interventions. Assessment is the first fundamental step in the identification, prevention and management of adverse effects. Despite routine use of prospective, standardized clinical approaches to appraise immunosuppressive drug efficacy by health care providers, most adverse effects are not assessed in a systematic, objective manner. Therefore, we developed and evaluated the application, reliability and validity of a novel non-renal adverse effects assessment system in stable renal transplant recipients receiving maintenance immunosuppression consisting of a calcineurin inhibitor (cyclosporine or tacrolimus) and mycophenolic acid with or without prednisone.

## Methods

A novel immunosuppressive adverse effects assessment for 18 individual adverse effects (Tables 1 and 2) were developed by a medical sub-specialist group within the University at Buffalo Nephrology/Transplant Program. This adverse effect scoring system was evaluated in two prospective studies with the enrollment of 58 stable renal transplant recipients who participated in clinical pharmacology studies from 2007 to 2013. Stable patients at least 12 months post-renal transplant receiving maintenance immunosuppression consisting of either tacrolimus (*Prograf)* with enteric coated mycophenolate sodium (EC-MPS; *Myfortic)* or cyclosporine *(Neoral)* with mycophenolate mofetil (MMF; *CellCept*) were enrolled. Studies were approved by the University of Buffalo Human Subjects Institutional Review Board. All patients provided informed consent upon enrollment.

**Table 1:** Immunosuppressive adverse effects scoring system with gastrointestinal and aesthetic adverse effects

**Gastrointestinal adverse effects**^**a**^	
**Vomiting**	
0:	None
1+:	Minimal vomiting
2+:	Excessive vomiting requiring symptomatic treatment
**Diarrhea**	
0:	None
1+:	One loose bowel movement per day
2+:	Two to five loose bowel movements per day
**Dyspepsia**	
0:	None
1+:	Episode of indigestion within one hour after taking immunosuppressive medication
2+:	Indigestion for at least half the day
3+:	Indigestion the majority of the day requiring symptomatic treatment
**Acid suppressive therapy**	
0:	None
1+:	Daily use of a histamine-2 receptor antagonist OR a proton pump inhibitor
2+:	Daily use of a histamine-2 receptor antagonist AND a proton pump inhibitor
**Aesthetic adverse effects**^**a**^	
**Acne**	
0:	None
1+:	Lesion restricted to face, no specific treatment
2+:	Numerous facial lesions; lesions of upper trunk
3+:	Grade II and topical treatment
4+:	Grade II and systemic treatment
**Skin changes**	
0:	No change
1+:	No gross change but easy bruising
2+:	Obvious thinning multiple or frequent ecchymoses
3+:	Grade II and easy sloughing and/or striae; lacerations
**Hirsutism**	
0:	None
1+:	New hair growth anywhere
2+:	Marked increase in hair growth with noticeable change in appearance
3+:	Grade II and use of depilatories or shaves
**Moon facies**	
0:	No change
1+:	Rounding of jaws barely detected
2+:	Marked rounding with noticeable change in appearance
3+:	Grade II with plethora
**Gingival hyperplasia**	
0:	Absent
1+:	Some hyperplasia
2+:	Severe hyperplasia and bleeding
**Buffalo hump**	
0:	Absent
1+:	Present

^**a**^The scoring system presents a quantitative severity rating of the most common adverse effects of immunosuppressive therapy. Each adverse effect was assessed as a change from pre-transplant status to document that the manifestation was attributed to immunosuppressive therapy or a change related therapy in the post-transplant period.

**Table 2:** Immunosuppressive adverse effects scoring system with central nervous system and miscellaneous adverse effects

**Central nervous system adverse effects**^**a**^		
**Tremor**		
0:	None	
1+:	Noticeable movement of paper suspended over outstretched hands with fingers spread	
2+:	Prominent movement of suspended paper	
3+:	Resting tremor and/or tremor that impairs function	
**Headache**		
0:	Absent	
1+:	Present	
**Insomnia**		
0:	None	
1+:	Difficulty sleeping	
2+:	Noticeable loss of sleep; requires pharmacotherapy to achieve adequate sleep	
3+:	Severe sleep deprivation; difficulty sleeping despite pharmacotherapy	
**Miscellaneous adverse effects**^**a**^		
**Myopathy**		
0:	None	
1+:	Complaints of muscle weakness or decreased strength	
2+:	Clinically apparent weakness difficulty rising from chair or squatting position	
3+:	Muscle wasting + severe limitation of exercise capacity	
**Ophthalmic changes**		
0:	No cataract formation	
1+:	Cataracts present; or surgical removal of cataracts has occurred	
**Mania/Excitable behavior**		
0:	None	
1+:	Excitable behavior; rapid speech and activity level	
2+:	Very excitable behavior, actions and speech	
**Depression**		
0:	None	
1+:	Depressed thoughts with normal level of activity for patient ( no antidepressants)	
2+:	Depressed thoughts with normal level of activity for patient ( with antidepressants)	
3+:	Depressed thoughts without normal level of activity for patient ( does not dress, poor hygiene, etc.) and receives anti-depressants	
**Post-transplant Diabetes mellitus**	Symptoms of diabetes plus casual plasma glucose ≥200 mg/dl	**OR**
	Fasting plasma glucose ≥126 mg/dl	**OR**
(two of the criteria are required)	2 hour plasma glucose ≥200 mg/dl during an oral glucose tolerance test	**OR**
0: Absent	Glycated Hemoglobin A1C > 7%	**OR**
1+: Present	Use of anti-diabetic agents	
**General laboratory results**		
Renal function – Serum Creatinine (mg/dl)	_____________________	
Blood pressure	_____________________	
Glucose (mg/dl)	_____________________	
Glycated hemoglobin A1C (%)	_____________________	
Total cholesterol (mg/dl)	_____________________	
High density lipoprotein (mg/dl)	_____________________	
Low density lipoprotein (mg/dl)	_____________________	
Triglycerides (mg/dl)	_____________________	
Total white blood cells (cell/mm^3^)	_____________________	
Neutrophils (%)	_____________________	
Lymphocytes (%)	_____________________	
Platelets (cells/mm^3^)	_____________________	
Hemoglobin (g/dl)	_____________________	
Hematocrit (%)	_____________________	

^**a**^The scoring system presents a quantitative severity rating of the most common adverse effects of immunosuppressive therapy. Each adverse effect was assessed as a change from pre-transplant status to document that the manifestation was attributed to immunosuppressive therapy or a change related therapy in the post-transplant period.

### Validity assessment

Face validity, or appearance of the scoring system to measure the defined adverse effect parameter, was developed through nephrologist consultation with medical sub-specialists (i.e. gastroenterologist consultation for dyspepsia, vomiting and diarrhea) and then applied to the target population of renal transplant recipients [18].

Content validity, or measurement of essential components of immunosuppressive adverse effects (Tables 1 and 2) was determined by a group of nephrologists who were subject matter experts using standard recommendations [18]. Fourteen nephrologists were invited to respond to a survey using the standardized rating scales and document the clinical importance of each adverse effect for assessment in the renal transplant population. Nephrologists ranked individual adverse effects for clinical importance post-transplant using a scale of: 1 = no importance through 4 = significant importance. Descriptive statistics were determined with an *a priori* critical value of 3 (moderate importance) to achieve content validation [18].

The adverse effects scoring system was applied to each renal transplant recipient enrolled in two clinical studies using similar inclusion and exclusion criteria to assess construct validity, or the extent the scoring system measures each adverse effect described [18]. Adverse effect ratios (gastrointestinal, central nervous system [CNS], aesthetic and cumulative adverse effects) were compared between renal transplant recipients stabilized on either cyclosporine and MMF or tacrolimus and EC-MPS regimens since pre-established differences in adverse effect profiles have been reported [7,19-22]. Construct validity was determined by analyzing adverse effect differences between renal transplant recipients receiving these two immunosuppressive regimens using general linear modeling.

### Reliability assessment

Inter-rater reliability was evaluated in 32 stable renal transplant recipients treated with pre-established immunosuppressive regimens [23]. Nephrologists separately assessed each patient using the adverse effects scoring system during the same clinic visit. Intra-class correlation was generated from a pooled adverse effect score including the 18 adverse effects. The Kappa statistic was determined for each adverse effect to document inter-rater reliability and reported as an overall mean with standard deviation. Intra-class correlation ≥ 0.7 and Kappa statistic ≥ 0.6 were established *a priori* as desired endpoints since these scores represent substantial agreement between physician raters [23,24].

### Implementation of adverse effects scoring system

Nephrologists evaluated 18 adverse effects for each renal transplant patient during a scheduled clinic visit using objective clinical documentation including physical exam, review of systems and recent laboratory results. Medication adherence was assessed by the physician and pharmacist through patient interviews. Evaluation of the immunosuppressive adverse effects using the standardized scoring system was completed once (or twice during inter-rater reliability phase) during a single clinic visit for each patient in this cross-sectional study. Adverse effects were assessed as a change from pre-transplant status in an effort to identify the post-transplant development of immunosuppressive adverse effects. Participating nephrologists received training prior to utilizing this adverse effect scoring system. The scoring system focused on common non-renal adverse effects associated with immunosuppressive regimens of either cyclosporine or tacrolimus, mycophenolic acid and low dose glucocorticoids. After development of the adverse effects scoring system, nephrologists evaluated individual stable renal transplant recipients using these pre-established criteria with documentation of severity (i.e. 0 = no adverse effect; 1+ = mild to 3+ = severe manifestations). An overall adverse effect total was determined for each patient using the sum of individual adverse effect scores. The cumulative adverse effect ratio was then calculated as the quotient of each patient’s total score divided by the maximum score of all possible manifestations. This cumulative adverse effect ratio represented the number of adverse effects with corresponding severity rating. The gastrointestinal, CNS, and aesthetic ratios were generated *a posteriori* to further evaluate the organ system specific adverse effects. For example, if a patient received the following adverse effect evaluations: vomiting 0, diarrhea 1+, dyspepsia 1+, acid suppressive therapy 1+, the gastrointestinal adverse effect ratio would be 3/9 = 0.33 or 33% (9 is maximal possible score for gastrointestinal adverse effects). The denominator for each ratio provides normalization for the different severity rating among patients to allow comparison.

### Statistical analysis

Continuous data was presented as mean ± standard deviation and categorical data as frequency (percentage). Demographic and clinical parameters were evaluated using student’s T-test for parametric data, Wilcoxon for non-parametric data, or Chi-Square for categorical data. All statistical analysis was performed using SAS version 9.3 (SAS Institute, Cary NC).

## Results

A total of 58 stable renal transplant recipients were included in this study. Maintenance immunosuppressive regimens consisted of either cyclosporine and MMF (n = 30 males) or tacrolimus plus EC-MPS (n = 28 females and males). Table 3 provides a comparison of patient demographics for these two groups.

**Table 3:** Demographic characteristics

**Parameter**^**a**^	**Cyclosporine + MMF**	**Tacrolimus + EC-MPS**	**P value**
	**(n = 30)**	**(n = 28)**	
Age (years)	52.3 ± 9.29	54.1 ± 11.5	0.527^b^
Time post-transplant (years)	6.93 ± 4.27	5.15 ± 3.34	0.084^b^
Male	30 (100%)	13 (46.4%)	<0.001*
Caucasian	17 (56.7%)	13 (46.4%)	0.221^c^
BMI (kg/m^2^)	33.1 ± 6.73	30.4 ± 6.94	0.151^b^
eGFR (ml/min/1.73 m^2^)	51.7 ± 13.5	52.2 ± 18.7	0.921^b^
Albumin	4.24 ± 0.37	4.26 ± 0.41	0.839^b^
WBC (cells/mm^3^)	6.81 ± 2.26	6.38 ± 1.52	0.401^b^
MPA dose^e^	1417 ± 407	1242 ± 319	0.048^d^*
Prednisone use	12 (40.0%)	9 (32.1%)	0.585 ^c^
Calcineurin inhibitor trough concentration (ng/ml)	124 ± 47.1	6.80 ± 1.98	NA

^a^Data presented as mean ± standard deviation or frequency (percentage); ^b^T test; ^c^ Chi-square test; ^d^Wilcoxon test; ^e^Expressed as active mycophenolic acid.

*p ≤ 0.05: statistical significance.

BMI, body mass index; eGFR, estimated glomerular filtration rate; MMF, mycophenolate mofetil; MPA, mycophenolic acid; EC-MPS, enteric coated mycophenolate sodium; NA, not applicable; WBC, white blood cells.

Content validity was verified by 9 nephrologists out of the 14 requests for completion of the rating survey. The nephrologists’ survey confirmed that the adverse effects scoring system provided moderate-to-significant clinical importance (3.1 ± 0.75 with maximum score of 4.0) for the assessment of the 18 adverse effects in renal transplant recipients.

Table 4 provides severity scores and frequencies of individual adverse effects for the patients receiving the two immunosuppressive regimens. Post-transplant diabetes mellitus was observed in 30.0% of patients receiving cyclosporine and MMF compared to 32.1% of patient receiving tacrolimus (P = 0.860). Patients treated with tacrolimus and EC-MPS experienced higher gastrointestinal adverse effect ratio (0.21 ± 0.15) compared to cyclosporine and MMF, (0.12 ± 0.11; P = 0.008). Greater CNS adverse effect ratio was found in patients treated with tacrolimus and EC-MPS (0.26 ± 0.18) compared to cyclosporine and MMF (0.16 ± 0.17; P = 0.035). There was no difference between patients treated with tacrolimus and EC-MPS or cyclosporine and MMF for the cumulative (0.154 ± 0.069 vs. 0.128 ± 0.080; P = 0.197) or aesthetic (0.101 ± 0.089 vs. 0.133 ± 0.084; P = 0.161) adverse effect ratios, respectively. For individual adverse effects, 76.7% of patients treated with cyclosporine and MMF experienced gingival hyperplasia compared to 21.4% of those treated with tacrolimus and EC-MPS (P < 0.0001). Of the 316 individual adverse effects scored in all patients, 84.5% were rated as 1+; 14.9% were rated as 2+; and less than 1% rated as 3+ (Table 4).

**Table 4:** **Frequency of severity scores for immunosuppressive adverse effects**^**a**^

**Adverse effects**	**0**	**1+**	**2+**	**3+**	**Overall frequency (as %)**
Vomiting	30 / 27	0 / 1	0 / 0	NA	0 / 3.57
Diarrhea	28 / 20	2 / 8	0 / 0	NA	6.67 / 28.6
Dyspepsia	19 / 14	9 / 5	2 / 9	0 / 0	36.7 / 50.0
Acid suppressive therapy	12 / 9	18 / 16	0 / 3	NA	60.0 / 67.9
Acne ^b^	26 / 21	4 / 4	0 / 3	0 / 0	13.3 / 25.0
Skin changes	21 / 21	8 / 5	1 / 2	0 / 0	30.0 / 25.0
Hirsutism	22 / 22	7 / 3	1 / 3	0 / 0	26.7 / 21.4
Moon facies	13 / 17	15 / 11	2 / 0	0 / 0	56.7 / 39.3
Gingival hyperplasia	7 / 22	21 / 6	2 / 0	NA	76.7 / 21.4
Buffalo hump^c^	21 / 22	9 / 6	NA	NA	30.0 / 21.4
Tremor	13 / 7	15 / 16	2 / 4	0 / 1	56.7 / 75.0
Headache^c^	28 / 22	2 / 6	NA	NA	6.67 / 21.4
Insomnia	22 / 15	5 / 9	2 / 4	1 / 0	26.7 / 46.4
Myopathy	27 / 24	2 / 4	1 / 0	0 / 0	10.0 / 14.3
Ophthalmic changes^c^	17 / 18	13 / 10	NA	NA	43.3 / 35.7
Mania	29 / 28	1 / 0	0 / 0	NA	3.33 / 0
Depression	26 / 18	4 / 4	0 / 6	0 / 0	13.3 / 35.7
Post-transplant diabetes mellitus^c^	21 / 19	9 / 9	NA	NA	30.0 / 32.1

Data displayed as cyclosporine and MMF regimen (n = 30) / tacrolimus and EC-MPS regimen (n = 28).

^a^Severity score determined using Tables 1 and 2; ^b^No patients received a 4+ for acne; ^c^ rated as present or absent.

NA, non-applicable since no rating; MMF, mycophenolate mofetil; EC-MPS, enteric coated mycophenolate sodium.

**Figure 1:**
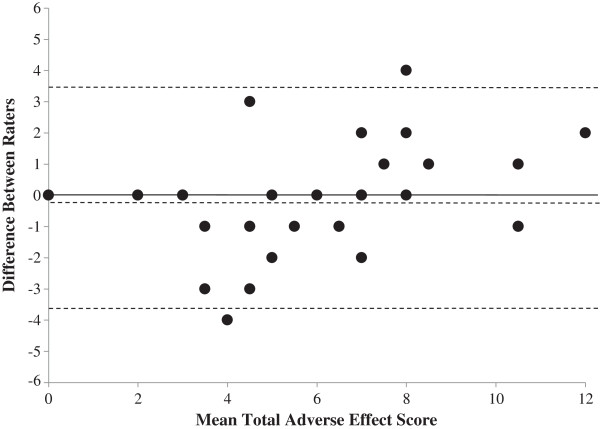
**Bland-Altman Plot for Immunosuppressive Adverse Effects Scoring System.** This figure presents the inter-rater agreement of n = 32 renal transplant recipients for the adverse effects scoring system with the difference between raters on the y-axis and the mean total adverse effect score for each patient on the x-axis. Each solid dot represents one patient (Note: there are 7 overlapping data points). The dotted lines represent the mean difference between the two raters (−0.15; middle line) with the upper and lower bounds of 2 standard deviations around the mean (−3.59, +3.28). There is little systematic bias or variability between raters.

The mean Kappa statistic was 0.68 ± 0.25. The intra-class correlation was 0.81 (95% confidence interval [CI]: 0.65-0.90) to represent the reliability of summary scores for the 18 adverse effects. The intra-class correlations for adverse effect ratios exceeded the *a priori* designation as follows: 0.75 (CI: 0.55-0.87) for cumulative; 0.78 (CI: 0.60-0.88) for gastrointestinal; 0.81 (CI: 0.65-0.90) for CNS; and 0.84 (CI: 0.70-0.92) for aesthetic adverse effect ratios. Inspection of the Bland-Altman graph (Figure 1) revealed limited variability between raters without systematic bias.

## Discussion

This study presents a novel standardized immunosuppressive adverse effects scoring system for renal transplant recipients with validation and inter-rater reliability demonstrated. Multidisciplinary development of this scoring system with successful application to the intended population provides face validity [18]. The heterogeneous nature of the individual adverse effects depicted in Table 3 emphasizes varying degrees of severity and frequency among stable renal transplant recipients receiving calcineurin inhibitor based immunosuppressive regimens. Severity assessment of a 3+ score for any adverse effect was less than 1% of all adverse effects during this study since clinically stable patients were targeted for enrollment. Identifying transplant patients with mild to moderate adverse effects may have important clinical impact since drug regimen adjustments during maintenance immunosuppression may improve or eliminate these manifestations. Renal transplant recipients with numerous mild to moderate adverse effects also have increased risk for medication non-adherence as time post-transplant increases which may impact long-term allograft survival [16,17]. Therefore, prospective, standardized monitoring of immunosuppressive adverse effects may reduce medication non-adherence, improve patient tolerability of immunosuppressive regimens and may improve renal allograft survival [16,17]. In addition, this adverse effects evaluation system provides an objective approach to document and rank severity of medication related effects in patients during clinic visits. Differences in the rating scales for the individual adverse effects are present (i.e. 0-2+, 0-3+) based on the smallest detectable incremental change in manifestation clinically observed by the physician. This rating is adjusted by using the cumulative and organ system specific adverse effect ratios which incorporates internal normalization in order to compare patients. This approach documents adverse effect frequency and severity which is rarely verified in clinical studies [21].

Nephrologists as subject matter experts verified moderate to significant clinical importance for the individual adverse effects to be used in the scoring system and substantiated content validity [18]. Some limitations exist with this evaluation including physician bias with current prescribing trends for low glucocorticoid doses or steroid-free immunosuppressive regimens post-transplant. These prescribing trends may account for lower clinical importance scores of steroid associated adverse effects. Since interpatient variation in clinical response, drug exposure, and adverse effect manifestations to steroid therapy post-transplant exists, these adverse effects will remain in the scoring system to guide maintenance immunosuppression [25-27]. Tailoring the specific adverse effects scoring system to reflect the inclusion or exclusion of glucocorticoids (i.e. prednisone) in the immunosuppressive regimen provides a practical and flexible objective monitoring alternative for transplant recipients.

The adverse effect ratios documented that renal transplant recipients receiving tacrolimus and EC-MPS regimen had greater gastrointestinal and CNS adverse effects compared to patients stabilized on the cyclosporine and MMF regimen. These findings are consistent with previous clinical reports [7,22]. Aesthetic adverse effects such as gingival hyperplasia, hirsutism, acne, and skin changes are more frequent in cyclosporine treated patients, but was not observed in this study for the aesthetic adverse effect ratio [7,21]. This finding may be attributed to sample size or the male predominance in the cyclosporine group. Additionally, individual aesthetic adverse effects such as gingival hyperplasia was more frequent in the cyclosporine treated patients and confirms adverse effect patterns observed in renal transplant recipients participating in comparative efficacy studies between these two immunosuppressive regimens. This finding further verifies construct validity [19,20,22]. The cumulative adverse effect ratio was created *a priori* with incorporation of the normalization for purpose of interpatient comparison. No differences were noted in the cumulative ratio between regimens. However, gastrointestinal, central nervous system (CNS), and aesthetic adverse effect ratios were generated *a posteriori* to further quantitate organ system specific adverse effects and interpatient differences of these manifestations were successfully demonstrated. Potential construct limitations include confounding factors such as concomitant medications with overlapping adverse effect profiles and the inability to distinguish causative relationships to individual medications. Since this scoring system provides a composite of adverse effects manifested by common immunosuppression regimens, additional factors such as concomitant medications or co-morbidities should also be considered during routine clinical assessment.

Inter-rater reliability of the scoring system was substantial based on intra-class correlation and Kappa analyses documenting agreement between raters [23,24]. The overall scoring system and each organ system adverse effect ratio achieved the *a priori* endpoint for intra-class correlation ≥ 0.7. Qualitative assessment of specificity was observed through rigorous patient assessment methodologies to characterize the adverse effects, the significant inter-rater reliability, and calcineurin inhibitor specific differences in adverse effect profiles observed.

The adverse effects scoring system administered by a clinician provides a time efficient, standardized clinical approach to assess the frequency and severity of these manifestations in renal transplant recipients. In contrast, the Memphis survey provides a patient completed questionnaire with comprehensive evaluation of symptoms and quality of life post-transplant, but does not provide succinct, efficient, objective adverse effects evaluation completed by the practitioner [28]. In addition, the patient completed gastrointestinal symptom rating scale validated for renal transplant recipients does not include physician verification or other organ-specific adverse effects [29]. Therefore, routine clinical application of these patient rating scales may be limited for objective immunosuppressive monitoring.

This is a single-center experience in stable renal transplant recipients receiving maintenance immunosuppression according to pre-established protocols. Therefore, variability observed in adverse effect manifestations as detected by the scoring system supports internal validity. As a single center experience, consideration of scoring bias by participating nephrologists who may provide routine medical care to some of these patients was possible. Due to the high inter-rater reliability, this type of bias was minimized. The novelty of the scoring system is the utilization of clinician directed evaluation of common adverse effects using a systematic, objective approach instead of a reactive response to these manifestations. A few infrequent adverse effects (e.g. alopecia) were not included and may be considered a study limitation.

Future research using this validated adverse effect rating system may include longitudinal evaluation of immunosuppressive adverse effects during the acute and chronic post-transplant periods and relationships to medication adherence, physician evaluation and allograft outcomes. This preliminary report provides a novel standardized adverse effect assessment which may benefit other solid organ transplant populations through prospective evaluations.

## Conclusions

This validation study presents a clinician administered immunosuppressive adverse effects scoring system for stable renal transplant recipients which provides face, content and construct validity with inter-rater reliability. The scoring system provides a systematic, standardized, objective assessment of immunosuppressive adverse effects that could be utilized for periodic evaluation (i.e. quarterly) post-transplant to enhance individualization of immunosuppression. This approach to clinical adverse effects monitoring may improve patient safety and immunosuppressive adherence post-transplant.

## Abbreviations

BMI: Body mass index; CI: 95% Confidence interval; CNS: Central nervous system; EC-MPS: Enteric-coated mycophenolate sodium; eGFR: Estimated glomerular filtration rate; MMF: Mycophenolate mofetil; MPA: Mycophenolic acid; NA: Not applicable; WBC: White blood cells.

## Competing interests

This study was partially supported by an Investigator Initiated Research Grant from Novartis Pharmaceuticals with Dr. Tornatore as the principal investigator and Dr. Venuto as a co-investigator. Other authors have no financial involvements to disclose.

## Authors’ contributions

All authors contributed to the design and conduct of the study. CJM, KMT, RCV and ZA contributed to the patient enrollment and consents. The adverse effects in patients were assessed by ZA and RCV who also contributed to data summary with CJM and KMT. JC and GW conducted the statistical analyses and contributed to the Methods section for this manuscript. All authors reviewed and contributed to data interpretation and manuscript development. CJM and KMT wrote the manuscript based upon review of results, clinical implications and edits provided by co-authors. All authors completed review and editing of this manuscript.

## Pre-publication history

The pre-publication history for this paper can be accessed here:

http://www.biomedcentral.com/1471-2369/15/88/prepub
